# Antimicrobial Resistance, Virulence Determinants, and Biofilm Formation of *Enterococcus* Species From Ready-to-Eat Seafood

**DOI:** 10.3389/fmicb.2019.00728

**Published:** 2019-04-18

**Authors:** Etinosa O. Igbinosa, Abeni Beshiru

**Affiliations:** ^1^Applied Microbial Processes and Environmental Health Research Group, Department of Microbiology, Faculty of Life Sciences, University of Benin, Benin City, Nigeria; ^2^Sustainable Development Office, University of Benin, Benin City, Nigeria

**Keywords:** shrimp varieties, enterococci, antibiotic-resistant, biofilm producers, risk assessment

## Abstract

*Enterococcus* species form an important population of commensal bacteria and have been reported to possess numerous virulence factors considered significantly important in exacerbating diseases caused by them. The present study was designed to characterize antibiotic-resistant and virulent enterococci from ready-to-eat (RTE) seafood. A total of 720 RTE shrimp samples comprising sauced shrimp (*n* = 288), boiled shrimp (*n* = 216), and smoked shrimp (*n* = 216) obtained from open markets in Delta State, Nigeria, were assessed. Standard classical methods and polymerase chain reaction (PCR) were used in identifying the *Enterococcus* species. Potential virulence factors (β-hemolysis, gelatinase activity, S-layer, and biofilm formation) were assessed using standard procedures. The antibiotic susceptibility profile of the identified enterococci isolates was assayed using the Kirby–Bauer disc diffusion method. PCR was further used to screen selected antibiotic resistance and virulence genes. Prevalence of *Enterococcus* species from shrimp varieties is as follows: sauced, 26 (9.03%); boiled, 6 (2.78%); and smoked, 27 (12.50%), with an overall prevalence of 59 (8.19%) based on the occurrence of black hallow colonies after incubation. *Enterococcus* species detected include *E. faecalis*, 17 (28.8%); *E. faecium*, 29 (49.2%); *E. gallinarum*, 6 (10.2%); *E. casseliflavus*, 2 (3.4%); *E. hirae*, 3 (5.1%); and *E. durans*, 2 (3.4%). Biofilm occurrence among the shrimp varieties is as follows: 19/26 (73.1%) for sauced shrimps, 5/6 (83.3%) for boiled shrimps, and 16/27 (59.3%) for smoked shrimps. The phenotypic expression of the enterococci virulence revealed the following: S-layer, 59 (100%); gelatinase production, 19 (32.2%); and β-hemolysis, 21 (35.6%). An average of 3–11 virulence genes were detected in the *Enterococcus* species. The resistance profile of *Enterococcus* species is as follows: erythromycin, 29 (49.2%); vancomycin, 22 (37.3%); and tetracycline, 27 (45.8%). The frequency of occurrence of antibiotic resistance genes from the phenotypic resistant enterococci isolates to the macrolide, glycopeptide, and tetracycline antibiotics is as follows: *erm*A, 13/29 (44.8%); *van*A, 14/22 (63.6%); *tet*A, 14/27 (51.9%); *tet*M, 15/27 (55.6%); *erm*B, 4/29 (13.8%); and *van*B, 5/22 (22.7%). Findings from this study reveal the antibiotic resistance of enterococci strains of such species as *E. durans, E. casseliflavus, E. gallinarum*, and *E. hirae*. This study further revealed that RTE food products are reservoirs of potential virulent enterococci with antibiotic-resistant capabilities. This provides useful data for risk assessment and indicates that these foods may present a potential public health risk to consumers.

## Introduction

The world shrimp production for both farmed and captured shrimp is approximately six million tons, with 60% entering the world market. Shrimp has been reported to be the most essentially traded fishery product internationally as it translates to value. Yearly shrimp exports presently value above US $10 billion, or 16% of total fish product exports (Food and Agriculture Organization of the United Nations [FAO], 2008). Shrimp makes up 20% of exported fishery products for more than 20 years (CAC, 2002). Imports of shrimps into developed nations are responsible for about 40% of trade in intra-developed countries, while approximately 60% comes from developing nations. From developing nation exports, 80% goes to developed nations with only 20% left behind (Josupeit, 2005). Shrimps are one of the important exported aquaculture products from the tropics.

*Enterococcus* species are found commonly in the gastrointestinal tract of farmed animals and humans. They form an essential population of commensal bacteria encompassed in the functional microbiota (Lebreton et al., 2014). Epidemiological and ecological findings specify that, along with fecal matter, these commensal bacteria are released into environs they colonize easily as a result of their high adaptability. This translates to their disseminated occurrence in water, soil, sewage, fruits, and plants. Through this means, they consequently exist in raw materials of plant and animal origin (meat, vegetables, and milk) (Giraffa, 2002). *Enterococcus* spp. occurrence in foods, with ready-to-eat (RTE) food type inclusive, occurs particularly from their adaptability to severe conditions in the environment as it relates to production and storage conditions. The capability of *Enterococcus* species to proliferate in the occurrence of NaCl (5–10%), bile salt (40%), pH range (4.6–9.9), and anaerobic and aerobic conditions, and its capacity to survive for 30 min in a temperature of 63.5°C revealed that they usually account for the lingering microflora in RTE food (Van-den-Berghe et al., 2006).

Enterococci have been implicated in serious infections, reduced production in the grow-out ponds and hatchery, and decreased growth rates and feed conversion in surviving individuals, thereby eliciting a negative impact on the complete financial productivity of the business (El-Far et al., 2015). Most pathogenesis of infectious enterococci comprises series of events such as establishment, adhering to the host’s cell structure, tissue invasion, and unspecific resistance defense mechanism (Upadhyaya et al., 2009). Literature has reported enterococci that expressed virulence phenotypically to express infections severely compared to those that did not express them at all. The elevated mechanism of enterococci as causative agents that cause infections in patients having their immunity compromised has invigorated further investigations to characterize the elements and/or factors that allow bacteria to inhabit the host effectively through the barriers of the immune system causing pathological alterations (Chajecka-Wierzchowska et al., 2017).

Continuous proliferation in the amount of *Enterococcus* strains that are antimicrobial resistant has been documented (Carlet et al., 2012). Vancomycin-resistant *Enterococcus* species presently accounts for the foremost opportunistic pathogen in nosocomial environments (O’Driscoll and Crank, 2015). As such, an upsurge in literature that concerns the mechanisms of resistance of these bacteria to several antibiotics has occurred. Occurrence of resistance determinants only does not signify strain pathogenesis. Conversely, when in conjunction with the occurrence of virulence potentials, it may result in dangerous strains (Heidari et al., 2016). This occurs in particular since genes expressing/conveying resistance virulence factors/elements and antibiotics are usually sited on similar or same genetic determinants. Antibiotic resistance plasmids, transposons, and virulence genes with transmissible characteristics have been reported in literature to be transmissible via highly proficient mechanisms of gene transfer (Eaton and Gasson, 2001).

Enterococci are described as vital hospital-associated pathogens and have thus been reported to withhold lots of virulence potentials considered significantly essential in exacerbating ailments caused by them. *Enterococcus* strains of clinical origin have been described extensively in literature with limited information of the phenotypic virulence factors coupled with its genetic structure from RTE seafood. Furthermore, enterococci have demonstrated intrinsic antimicrobial resistance to numerous antibiotic agents and can adapt to obtain resistance to antimicrobials from the environment (Beshiru et al., 2017). Multiresistance to a diverse class and subclass of antibiotics along with occurrence of virulence potentials strengthens the vital roles of *Enterococcus* species as opportunistic pathogens. Vancomycin-resistant *Enterococcus faecium* have increasingly been reported since the 1980s. Despite the significant number of literature about vancomycin-resistant *Enterococcus* epidemiology, the evolution and dynamics of these microorganisms are yet to be fully understood. Freitas et al. (2016) reported that both plasmids and strains contributed to the persistence and spread of vancomycin resistance among *E. faecium*. Horizontal gene transfer from different clonal lineages (different or same species) results in chimeras with dissimilar host range and stability, thwarting the surveillance of epidemic plasmids.

Outbreaks of *Enterococcus* species from hospital and environmental sources have been reported in literature in recent times (Gassiep et al., 2015; Lister et al., 2015; O’Driscoll et al., 2015; Pinholt et al., 2015; Sivertsen et al., 2016; Ulrich et al., 2017) with little or no information on outbreaks of enterococci infection of food origin. However, consumption of RTE food conveying virulence potentials is an important route of transfer (Chajecka-Wierzchowska et al., 2017). The current study was carried out to characterize antibiotic-resistant and virulent enterococci from RTE seafood.

## Materials and Methods

### Sample Collection and Isolation

Seven hundred and twenty RTE shrimp samples were obtained from markets in Delta State, Nigeria. The different markets sampled from Delta State and their respective coordinates coupled with the sampling procedure have been described previously by Beshiru et al. (2018). A total of six different open markets were sampled, with a total of 60 samples from each market within a 12-month sampling duration. Ten shrimp samples were obtained from each market (four sauced, three boiled, and three smoked shrimps) monthly. The total distribution of the different varieties of shrimp sampled in Delta State is as follows: sauced shrimp, *n* = 288; boiled shrimp, *n* = 216; and smoked shrimp, *n* = 216.

Twenty-five grams of each RTE shrimp sample was homogenized in 225 mL of sterile tryptone soy broth (Lancashire, United Kingdom) and incubated overnight at 37°C. A streak plate method was adopted via streaking from the overnight broth cultures on bile esculin agar (Darmstadt, Germany), which were incubated for 24 h at 37°C. Two to three distinct colonies with black hallow characteristics on the bile esculin agar were purified on nutrient agar (Lancashire, United Kingdom) and incubated for 24 h at 37°C. Purified isolates were stored on nutrient agar slants and maintained at 4°C in the refrigerator.

### Identification of *Enterococcus* Species

Gram-positive cocci were characterized further using the Analytical Profile Index 20E (BioMerieux, Marcy-l’Etoile, France) in accordance with the instructions of the manufacturer. *Enterococcus faecalis* (ATCC 19433) was used as a positive control. Strips were examined accordingly and identification was secured via an API lab plus software (BioMerieux, Marcy l’Etoile, France). Isolates screened using API were thereafter subjected to DNA extraction using the modified boiling method (Beshiru et al., 2017). PCR using genus-specific and species-specific primers in Table 1 and PCR conditions previously described was used in the identification of the *Enterococcus* species (Dutka-Malen et al., 1995; Deasy et al., 2000; Kariyama et al., 2000; Jackson et al., 2004). Positive controls used include *E. faecalis* (ATCC 19433), *E. faecium* (ATCC 19434), *Enterococcus casseliflavus* (ATCC 25788), *Enterococcus durans* (ATCC 19432), *Enterococcus hirae* (ATCC 8043), and *Enterococcus gallinarum* (ATCC 700425). For the negative control, deionized water was used in place of the template for each test procedure. The Peltier-Based Thermal Cycler (MG96Þ/Y, Zhejiang China) was used in the amplification process. Electrophoresis of the PCR amplicons was performed with 1.5% agarose gel (CLS-AG100, Warwickshire, United Kingdom) in 0.5× TAE buffer (40 mM Tris-HCl, pH 8.5, 1 mM EDTA, and 20 mM Na acetate) and allowed to run for 55 min at 100 V. The gels are visualized under a UV transilluminator (EBOX VX5, Vilber Lourmat, France).

**Table 1:** Primers used in the study.

Species/gene targeted	Primer sequence	Amplicon size (bp)	References
*Enterococcus* spp.	TCA ACC GGG GAG GGT ATT ACT AGC GAT TCC GG	733	Deasy et al., 2000
*E. faecalis*	TCA AGT ACA GTT AGT CTT TAT TAG ACG ATT CAA AGC TAA CTG AAT CAGT	941	Dutka-Malen et al., 1995
*E. faecium*	TTG AGG CAG ACC AGA TTG ACG TAT GAC AGC GAC TCC GAT TCC	658	Dutka-Malen et al., 1995
*E. casseliflavus*	CGG GGA AGA TGG CAG TAT CGC AGG GAC GGT GAT TTT	488	Kariyama et al., 2000
*E. gallinarum*	GGT ATC AAG GAA ACC TC CTT CCG CCA TCA TAG CT	822	Kariyama et al., 2000
*E. durans*	CCT ACT GAT ATT AAG ACA GCG TAA TCC TAA GAT AGG TGT TTG	295	Jackson et al., 2004
*E. hirae*	CTT TCT GAT ATG GAT GCT GTC TAA ATT CTT CCT TAA ATG TTG	187	Jackson et al., 2004
*cyl*A	ACT CGG GGA TTG ATA GGC GCT GCT AAA GCT GCG CTT	688	Vankerckhoven et al., 2004
*Ace*	AAA GTA GAA TTA GAT CCA CAC TCTA TCA CAT TCG GTT GCG	320	Mannu et al., 2003
*efa*A	CGT GAG AAA GAA ATG GAG GA CTA CTA ACA CGT CCA CGA ATG	499	Mannu et al., 2003
*gel*E	AGT TCA TGT CTA TTT TCAC CTT CAT TAT TTA CAC GT TTG	402	Mannu et al., 2003
*Agg*	CCA GTA ATC AGT CCA GAA ACA AACC TAG CTT TTT TCA TTC TTG TGT TTG TT	406	Mannu et al., 2003
*Esp*	TTA CCA AGA TGG TTC TGT AGG CAC CCA AGT ATA CTT AGC ATC TTT TGG	913	Shankar et al., 1999
*Cpd*	TGG TGG GTT ATT TTT CAA TTC TAC GGC TCT GGC TTA CTA	782	Eaton and Gasson, 2001
*Ccf*	GGG AAT TGA GTA GTG AAG AAG AGC CGC TAA AAT CGG TAA AAT	543	Eaton and Gasson, 2001
*Cob*	AAC ATT CAG CAA ACA AAGC TTG TCA TAA AGA GTG GTCAT	1405	Eaton and Gasson, 2001
*cyl*L	GATGGAGGGTAAGAATTATGG GCTTCACCTCACTAAGTTTTATAG	253	Semedo et al., 2003
*Hyl*	ACAGAAGAGCTGCAGGAAATG GACTGACGTCCAAGTTTCCAA	276	Vankerckhoven et al., 2004
*sprE*	TTGAGCTCCGTTCCTGCCGAAAGTCATTC TTGGTACCGATTGGGGAACCAGATTGACC	591	Nakayama et al., 2001
*mph*C	GAGA CTAC CAAG AAGA CCTGACG CATA CGCC GATT CTCC TGAT	722	Lüthje and Schwarz, 2006
*van*B	AGACATTCCGGTCGAGGAAC GCTGTCAATTAGTGCGGGAA	220	Nam et al., 2012
*van*A	GCGCGGTCCACTTGTAGATA TGAGCAACCCCCAAACAGTA	314	Nam et al., 2012
*van*C-2/3	CTAGCGCAATCGAAGCACTC GTAGGAGCACTGCGGAACAA	582	Nam et al., 2012
*van*C-1	ATCCAAGCTATTGACCCGCT TGTGGCAGGATCGTTTTCAT	402	Nam et al., 2012
*erm*A	GCGGTAAACCCCTCTGAG GCCTGTCGGAATTGG	434	Werckenthin and Schwarz, 2000
*erm*B	CATT TAAC GACG AAAC TGGC GGAA CATC TGTG GTAT GGCG	425	Jensen et al., 1999
*erm*C	ATCT TTGA AATC GGCT CAGG CAAA CCCG TATT CCAC GATT	295	Jensen et al., 1999
*tet*M	GTGGACAAAGGTACAACGAG CGGTAAAGTTCGTCACACAC	406	Ng et al., 2001
*tet*A	GCT ACA TCC TGC TTG CCTTC CAT AGA TCG CCG TGA AGAGG	210	Ng et al., 2001
*tet*B	TTG GTTA GGG GCA AGT TTTG GTA ATG GGC CAA TAA CACCG	659	Ng et al., 2001
*tet*C	CTTGAGAGCCTTCAACCCAG ATGGTCGTCATCTACCTGCC	418	Ng et al., 2001
*tet*D	AAA CCA TTAC GGCA TTC TGC GAC CGG ATA CAC CAT CCATC	787	Ng et al., 2001

### Antibiotic Susceptibility Profile of *Enterococcus* Isolates

The antimicrobial susceptibility profile of the *Enterococcus* species was determined using the Kirby–Bauer disc diffusion method. Briefly, the purified isolates were inoculated in 5.0 mL of Mueller–Hinton Broth (MHB; Lab M, Lancashire, United Kingdom) and incubated overnight. The optical density (OD) of the turbidity of the broth was determined to conform to the OD 0.5 of the McFarland standard where the cells are equivalent to 10^6^ cfu/mL. Using sterile swab sticks, the respective broth cultures were aseptically swabbed on Mueller–Hinton Agar (Lab M, Lancashire, United Kingdom). A total of 14 antibiotic discs (Mast Diagnostics, United Kingdom) that include penicillin G (10 units), piperacillin(100 μg), clindamycin (2 μg), vancomycin (30 μg), teicoplanin (30 μg), erythromycin (15 μg), tetracycline (30 μg), tigecycline (15 μg), kanamycin (30 μg), imipenem (10 μg), norfloxacin (10 μg), ciprofloxacin (5 μg), chloramphenicol (30 μg), and rifampin (5 μg) were used for susceptibility testing. The respective discs were also aseptically impregnated on the agar plates using sterile forceps spaced equidistant apart. Plates were allowed to stand at 28 ± 2°C for 5 min to allow the media to absorb effectively and incubated at 37°C for 18–24 h. Characterization of the resistance, susceptibility, or intermediate profile of the isolates was elucidated by measuring the zone of inhibition and compared with a standard interpretative chart to determine the sensitive, intermediate, and resistance pattern of the isolates to the antibiotics used using the Clinical and Laboratory Standards Institute [CLSI] (2017) interpretative chart.

The multiple antibiotic resistance index (MARI) was calculated as previously described by Krumperman (1983). Multidrug resistance profile was described as resistance to a minimum of one antibiotic in a minimum of three antimicrobial classes (Magiorakos et al., 2012).

(1) $$\text{Multiple antibiotic resistance index}=\frac{\text{Number of}\;\text{antibiotics to which resistance occured}}{\text{Total number of antibiotics to which the isolates were tested}}$$

### Phenotypic Virulence and Biofilm Formation

The phenotypic virulence profile of the isolates was determined as previously modified by Beshiru and Igbinosa (2018). Colonies cultivated on tryptone soy agar (TSA; Merck, Darmstadt, Germany) were resuspended in 20 mL of TSB. The turbidity of this suspension was adjusted to 10^6^ cells/mL. Hemolytic activity was determined on a sheep blood agar plate. Lipase activity was elucidated on TSA. Gelatinase production was determined on gelatin medium. DNA-degrading activity was ascertained on DNase agar plates. The presence of surface-layer (S-layer) was assessed by streaking cultures on TSA plates, enhanced with 0.1 mg/mL Coomassie brilliant blue R 250 (Merck, Darmstadt Germany). All experiments were performed in triplicate.

Biofilm formation was assessed using the modified protocol of Igbinosa et al. (2017). Briefly, purified colonies of *Enterococcus* species were resuspended in 10 mL of tryptone soy broth, incubated at 37°C for 18 h, and centrifuged for 2 min at 12,000 rpm. The cell pellets were washed in phosphate-buffered solution (PBS) with their adherence properties determined via the wells of sterile 96-well polystyrene microtiter plates with sterile TSB broth as negative control. *E. faecalis* (ATCC 19433) was used as a positive control. The microtiter plates were incubated for 24 h at 37°C, washed with sterile PBS, allowed to dry at 28 ± 2°C, and stained with crystal violet for 30 min. The wells were washed again with sterilized deionized water and left to dry at room temperature. Crystal violet dye bound to adherent cells was resuspended in 150 mL of 99% ethanol. The OD readings from respective wells were determined at 570 nm via a microtiter plate reader (Synergy Mx Biotek^®^, United States). Each assay was determined three times to obtain the mean value. Biofilm formation was characterized as a negative, weak, moderate, or strong producer in accordance with methods previously described by Basson et al. (2007).

### Antibiotic Resistance and Virulence Gene Determination

Macrolide-resistant genes (*mph*C, *erm*C, *erm*B, and *erm*A) were detected via PCR following the procedure of Sauer et al. (2008) using primers presented in Table 1. PCR program conditions include an initial denaturation step for 5 min at 94°C followed by 30 cycles, which includes denaturation for 60 s at 94°C, with the following respective annealing temperature regimen: *erm*A (51°C), *erm*B (51°C), *erm*C (51°C), and *mph*C (55°C) for 60 s, and extension for 60 s at 72°C with a final extension for 5 min at 72°C in one cycle, which ended the procedure. PCR conditions for *van*A, *van*B, *van*C-1, and *van*C-2/3 include a denaturation step of 94°C for 3 min, 35 cycles of denaturation at 94°C for 1 min, annealing at 56.5°C for 1 min, extension at 72°C for 1 min, followed by an elongation step at 72°C for 10 min. PCR conditions for *tet*A, *tet*B, *tet*C, *tet*D, and *tet*M genes include an initial denaturation for 5 min at 94°C, followed by 10 cycles of denaturation for 30 s at 94°C; annealing for 30 s at 58°C for *tet*A, *tet*B, *tet*C, and *tet*D; annealing for 30 s at 57°C for *tet*M; and extension for 45 s at 72°C. The Peltier-Based Thermal Cycler (MG96Þ/Y, Hangzhou, Zhejiang China) was used in the amplification process. Electrophoresis of the PCR amplicons was performed with 1.5% agarose gel (CLS-AG100, Warwickshire, United Kingdom) in 0.5× TAE buffer (40 mM Tris-HCl, pH 8.5, 1 mM EDTA, and 20 mM Na acetate) and allowed to run for 1 h at 100 V. The gels are visualized under a UV transilluminator (EBOX VX5, Vilber Lourmat, France).

Virulence genes such as enterococcal surface protein (*esp*), serine protease (*spr*E), gelatinase (*gel*E), enterococcal surface adhesion (*ace*), enterococcal cytolysin (*cyl*L), cytolysin operon (*cyl*A), aggregation substance (*agg*), sex pheromones (*ccf, cob*, and *cpd*), hyaluronidase (*hyl*), and cell wall adhesins (*efa*A) were amplified via PCR using specific primers in Table 1 and conditions previously described (Shankar et al., 1999; Eaton and Gasson, 2001; Nakayama et al., 2001; Mannu et al., 2003; Semedo et al., 2003; Vankerckhoven et al., 2004). The Peltier-Based Thermal Cycler (MG96Þ/Y, Hangzhou, Zhejiang, China) was used in the amplification process. Electrophoresis of the PCR amplicons was performed with 1.5% agarose gel (CLS-AG100, Warwickshire, United Kingdom) in 0.5× TAE buffer (40 mM Tris-HCl, pH 8.5, 1 mM EDTA, and 20 mM Na acetate) and allowed to run for 1 h at 100 V. The gels are visualized under a UV transilluminator (EBOX VX5, Vilber Lourmat, France).

## Results

### Prevalence of *Enterococcus* Species From the Shrimps

Prevalence of *Enterococcus* species from shrimp varieties as shown in Table 2 is as follows: sauced, 26 (9.03%); boiled, 6 (2.78%); and smoked, 27 (12.50%). From the different markets sampled, the prevalence is as follows: ESM (*Enterococcus* from Sapele market), 7 (5.83%); EUMM (*Enterococcus* from Ughelli main market), 9 (7.50%); EOMA (*Enterococcus* from Ogbegonogo market, Asaba), 8 (6.67%); EAMA (*Enterococcus* from Ashafor market, Aniocha), 10 (8.33%); EIMN (*Enterococcus* from Igbudu market, Warri), 14 (11.67%); and MOI (*Enterococcus* from main market, Oleh, Isoko), 11 (9.17%). An overall prevalence of 59 (8.19%) was recorded from the shrimps.

**Table 2:** Prevalence of *Enterococcus* species from ready-to-eat shrimp variety.

		No. of samples examined	No. of positive samples (%)
Shrimp variety	Sauced	288	26 (9.03)
	Boiled	216	6 (2.78)
	Smoked	216	27 (12.50)
	Total	720	59 (8.19)
Market sampled	ESM	120	7 (5.83)
	EUMM	120	9 (7.50)
	EOMA	120	8 (6.67)
	EAMA	120	10 (8.33)
	EIMN	120	14 (11.67)
	MOI	120	11 (9.17)
	Total	720	59 (8.19)

*ESM, *Enterococcus* from Sapele market; EUMM, *Enterococcus* from Ughelli main market; EOMA, *Enterococcus* from Ogbegonogo market, Asaba; EAMA, *Enterococcus* from Ashafor market, Aniocha; EIMW, *Enterococcus* from Igbudu market, Warri; MOI, *Enterococcus* from main market, Oleh, Isoko.*

### Distribution Profile of Enterococci From the Shrimps

The distribution of *Enterococcus* species is as follows: *E. faecalis*, 17 (28.8%); *E. faecium*, 29 (49.2%); *E. gallinarum*, 6 (10.2%); *E. casseliflavus*, 2 (3.4%); *E. hirae*, 3 (5.1%); and *E. durans*, 2 (3.4%). Four different *Enterococcus* species (*E. faecium, E. faecalis, E. hirae*, and *E. gallinarum*) were recovered from sauced shrimps; two different *Enterococcus* species (*E. faecium* and *E. faecalis*) were recovered from boiled shrimps, while five different *Enterococcus* species (*E. casseliflavus, E. faecalis, E. durans, E. faecium*, and *E. hirae*) were recovered from smoked shrimps (Table 3).

**Table 3:** Phenotypic and genotypic virulence characterization of *Enterococcus* species from RTE shrimps varieties.

Isolate code	*Enterococcus* sp.	Phenotypic virulence	Genotypic virulence	Shrimp variety	Biofilm formation
ESM-1	*E. faecium*	S-layer, gel	*gel*E-*spr*E-*agg-efa*A-*cpd*-*cob-ccf-esp-hyl*	Sauced	Moderate
ESM-2	*E. faecalis*	S-layer	*agg-efa*A*-ace*-*cpd-cob-ccf-ccf-esp-hyl*	Boiled	Strong
ESM-3	*E. casseliflavus*	S-layer	*agg-ace*-*cpd-cob-ccf-esp*	Smoked	Negative
ESM-4	*E. faecium*	S-layer, β-hem, gel	*gel*E-*spr*E-*agg-efa*A*-ace*-*cpd-cob-ccf-esp-hyl*	Sauced	Moderate
ESM-5	*E. faecalis*	S-layer	*efa*A*-cpd-ace-cob-esp-hyl*	Smoked	Moderate
ESM-6	*E. casseliflavus*	S-layer	*cob-ace-ccf-esp*	Smoked	Moderate
ESM-7	*E. faecalis*	S-layer, gel	*spr*E-*agg-efa*A*-ace*-*cob-ccf-esp-hyl*	Sauced	Strong
EUMM-1	*E. faecalis*	S-layer, β-hem	*cyl*L-*agg-efa*A-*cpd-cob-ccf-hyl*	Smoked	Negative
EUMM-2	*E. faecium*	S-layer	*efa*A*-cob-ace-ccf-esp-hyl*	Sauced	Moderate
EUMM-3	*E. faecium*	S-layer, β-hem, gel-	*gel*E*-ace*-*spr*E-*cyl*L-*agg-efa*A-*cpd-cob-ccf-esp-hyl*	Smoked	Negative
EUMM-4	*E. hirae*	S-layer	*cpd-efa*A*-cob-ace-esp*	Sauced	Negative
EUMM-5	*E. faecalis*	S-layer, gel	*gel*E*-efa*A-*spr*E-*cob-ace-ccf-esp-hyl*	Boiled	Weak
EUMM-6	*E. faecium*	S-layer	*agg-efa*A*-ace-cpd-cob-ccf-esp-hyl*	Smoked	Moderate
EUMM-7	*E. faecium*	S-layer, β-hem	*cpd-efa*A*-cob-ace-ccf-esp-hyl*	Sauced	Weak
EUMM-8	*E. faecium*	S-layer, β-hem, gel-	*gel*E*-efa*A*-spr*E-*agg-ace-cob ccf-esp-hyl*	Smoked	Negative
EUMM-9	*E. faecium*	S-layer, β-hem	*cpd-efa*A*-ace-cob-ccf-esp-hyl*	Smoked	Moderate
EOMA-1	*E. faecalis*	S-layer, gel	*gel*E*-ace-spr*E-*agg-efa*A*-cpd-cob-ccf-esp*	Sauced	Moderate
EOMA-2	*E. faecalis*	S-layer	*cpd-efa*A*-ace-cob-ccf-esp*	Boiled	Strong
EOMA-3	*E. faecium*	S-layer	*cob-efa*A*-ccf-ace-esp-hyl*	Smoked	Weak
EOMA-4	*E. faecium*	S-layer, gel	*gel*E*-efa*A*-spr*E-*cpd-ace-cob-ccf-esp-hyl*	Sauced	Moderate
EOMA-5	*E. faecium*	S-layer, gel	*gel*E*-efa*A*-spr*E-*cpd-ace-cob-ccf-esp-hyl*	Smoked	Moderate
EOMA-6	*E. durans*	S-layer	*agg-esp-cpd-cob-ccf*	Smoked	Negative
EOMA-7	*E. faecium*	S-layer, gel	*gel*E*-ace-spr*E*-agg-efa*A*-cpd-cob-ccf-esp-hyl*	Boiled	Negative
EOMA-8	*E. gallinarum*	S-layer	*cpd-esp-cob-ccf*	Smoked	Moderate
EAMA-1	*E. gallinarum*	S-layer	*agg-esp-cpd-cob-ccf*	Smoked	Negative
EAMA-2	*E. faecium*	S-layer, β-hem, gel	*gel*E*- efa*A*-ace-spr*E-*cyl*L-*agg*-*cob-ccf-esp-hyl*	Smoked	Negative
EAMA-3	*E. faecalis*	S-layer, β-hem, gel	*gel*E*-efa*A*-spr*E-*agg-ace-cpd-cob-ccf-esp-hyl*	Sauced	Weak
EAMA-4	*E. faecium*	S-layer	*cob-efa*A*-ccf-ace-esp-hyl*	Smoked	Moderate
EAMA-5	*E. faecium*	S-layer	*agg-efa*A*-cpd-ace-cob-ccf-esp-hyl*	Smoked	Strong
EAMA-6	*E. faecalis*	S-layer	*cob-efa*A*-ccf-esp-ace-hyl*	Sauced	Weak
EAMA-7	*E. faecium*	S-layer, β-hem	*cpd-efa*A*-ace-cob-ccf-esp-hyl*	Smoked	Negative
EAMA-8	*E. faecium*	S-layer, β-hem, gel	*gel*E*-ace-spr*E-*agg-efa*A*-cpd-cob-ccf-esp-hyl*	Sauced	Moderate
EAMA-9	*E. gallinarum*	S-layer	*cob-esp-ccf*	Smoked	Moderate
EAMA-10	*E. faecium*	S-layer, β-hem	*agg-efa*A*-cpd-ace-cob-ccf-esp-hyl*	Sauced	Moderate
EIMW-1	*E. faecium*	S-layer	*agg-efa*A*-cob-ace-esp-hyl*	Sauced	Strong
EIMW-2	*E. faecium*	S-layer, gel	*gel*E-*spr*E*-efa*A*-agg*-*cpd-cob-ace-ccf-esp-hyl*	Sauced	Negative
EIMW-3	*E. faecalis*	S-layer	*agg-efa*A*-cob-ace-ccf-esp-hyl*	Sauced	Weak
EIMW-4	*E. faecalis*	S-layer, β-hem	*cyl*L-*agg-efa*A*-ace-cob-ccf-esp-hyl*	Smoked	Weak
EIMW-5	*E. faecium*	S-layer	*cob-efa*A*-ccf-ace-esp-hyl*	Smoked	Moderate
EIMW-6	*E. faecium*	S-layer, β-hem, gel	*gel*E*-ace-spr*E-*agg-efa*A*-cpd-cob-ccf-esp-hyl*	Smoked	Moderate
EIMW-7	*E. faecium*	S-layer, β-hem	*cob-efa*A*-ace-ccf-esp-hyl*	Sauced	Weak
EIMW-8	*E. faecalis*	S-layer	*agg-efa*A*-cob-ace-ccf-esp-hyl*	Boiled	Weak
EIMW-9	*E. faecium*	S-layer	*agg-efa*A*-cob-ace-ccf-esp-hyl*	Smoked	Moderate
EIMW-10	*E. faecium*	S-layer, β-hem, gel	*gel*E*-efa*A*-spr*E-*agg-ace-cpd-cob-ccf-esp-hyl*	Sauced	Negative
EIMW-11	*E. faecalis*	S-layer, β-hem	*cyl*L*-ace-agg*-*cpd-efa*A*-cob-ccf-esp-hyl*	Smoked	Negative
EIMW-12	*E. gallinarum*	S-layer	*cob-esp-ccf*	Sauced	Negative
EIMW-13	*E. gallinarum*	S-layer	*agg-esp-cpd-cob-ccf*	Sauced	Moderate
EIMW-14	*E. faecium*	S-layer, gel	*gel*E*-efa*A*-ace-spr*E-*agg*-*ccf-esp-hyl*	Sauced	Strong
MOI-1	*E. faecalis*	S-layer, β-hem	*agg-efa*A*-ace-cpd-cob-ccf-esp-hyl*	Smoked	Negative
MOI-2	*E. faecalis*	S-layer, β-hem	*agg-efa*A*-esp-cpd-cob-ccf-hyl*	Smoked	Weak
MOI-3	*E. faecium*	S-layer, β-hem, gel	*gel*E*-ace-spr*E-*agg-efa*A*-cpd-cob-ccf-esp-hyl*	Sauced	Negative
MOI-4	*E. durans*	S-layer	*agg-esp-cpd-cob-ccf*	Smoked	Moderate
MOI-5	*E. faecium*	S-layer, β-hem	*cyl*L*-ace-agg-efa*A*-cob-ccf-esp-hyl*	Sauced	Negative
MOI-6	*E. faecium*	S-layer	*cob-ace-efa*A*-ccf-esp-hyl*	Sauced	Moderate
MOI-7	*E. faecalis*	S-layer, β-hem, gel	*gel*E*-ace-spr*E-*agg-efa*A*-cob-ccf-esp-hyl*	Boiled	Moderate
MOI-8	*E. faecalis*	S-layer	*cpd-ace-cob-ccf-esp-hyl*	Sauced	Strong
MOI-9	*E. hirae*	S-layer	*cpd-esp-cob-ccf*	Sauced	Negative
MOI-10	*E. hirae*	S-layer	*agg-esp-cob-ccf*	Smoked	Negative
MOI-11	*E. gallinarum*	S-layer	*agg-ace-cpd-esp-cob*	Sauced	Weak

*ESM, *Enterococcus* from Sapele market; EUMM, *Enterococcus* from Ughelli main market; EOMA, *Enterococcus* from Ogbegonogo market, Asaba; EAMA, *Enterococcus* from Ashafor market, Aniocha; EIMW, *Enterococcus* from Igbudu market, Warri; MOI, *Enterococcus* from main market, Oleh, Isoko; β-hem, beta-hemolysis; gel, gelatinase activity.*

Biofilm formation of the *Enterococcus* species includes the following: non-formers, 19 (32%); weak formers, 11 (19%); moderate formers, 22 (37%); and strong formers, 7 (12%). Overall, 40 (67.8%) were biofilm formers. Biofilm occurrence among the shrimp varieties is as follows: 19/26 (73.1%) for sauced shrimps, 5/6 (83.3%) for boiled shrimps, and 16/27 (59.3%) for smoked shrimps (Table 3).

### Phenotypic and Genotypic Expression of Virulence Determinants in the Enterococci

The phenotypic expression of the enterococci isolates for virulence in this study as presented in Table 3 revealed the following: 59 (100%) for S-layer, 19 (32.2%) for gelatinase production, and 21 (35.6%) for β-hemolysis. The genotypic expression of virulence as presented in Table 3 is as follows: *gel*E, 18 (30.5%); *spr*E, 19 (32.2%); *cyl*L, 6 (10.2%); *agg*, 37 (62.7%); *cpd*, 36 (61.0%); *cob*, 58 (98.3%); *ccf*, 56 (94.9%); *efa*A, 46 (77.9%); *esp*, 58 (98.3%); *ace*, 47 (79.7%); and *hyl*, 44 (74.6%). An average of 3–11 virulence genes was detected in the *Enterococcus* species. None of the enterococci isolates was simultaneously positive to all tested virulence genes. Screened sex pheromone genes (*cpd, cob*, and *ccf*) were detected in 32 (54.2%) of the enterococci isolates. Furthermore, 52 (88.1%) enterococci isolates had both the *cob* and *ccf* genes. The *cyl*A gene was not detected in any of the enterococci. The *cyl*L gene was present in *E. faecalis* and *E. faecium*, which were isolated from five smoked and one sauced shrimp variety (Table 3).

No isolate produced lipase or was positive for DNA-degrading activity. All *gel*E gene-carrying enterococci phenotypically expressed the gelatinase activity. However, one of the isolates that phenotypically expressed the gelatinase activity lacked the *gel*E gene. All *cyl*L gene-carrying isolates expressed β-hemolytic activity. However, not all the isolates that phenotypically expressed the β-hemolytic activity harbored the *cyl*L gene (Table 3). From the *esp* gene detected, all the isolates phenotypically expressed the S-layer activity. However, one isolate with S-layer expressed phenotypically lacked the *esp* gene but harbored the *efa*A gene (Table 3). Although 40 (67.8%) of the isolates were biofilm formers phenotypically, all 40/40 (100%) of the biofilm producers harbored the *esp* gene and displayed S-layer characteristics, and 12/40 (30%) harbored the *gel*E gene. However, some of the isolates with the *esp* gene, *gel*E gene, and S-layer characteristics did not produce biofilm (Table 3).

### Antibiotic Susceptibility Profile of the *Enterococcus* Species

The resistance profile of *Enterococcus* species in Table 4 is as follows: penicillin G, 52 (88.1%); piperacillin, 23 (38.9%); clindamycin, 40 (67.8%); erythromycin, 29 (49.2%); vancomycin, 22 (37.3%); teicoplanin, 32 (54.2%); and tetracycline, 27 (45.8%).

**Table 4:** Antibiotic susceptibility profile of the *Enterococcus* species from shrimps.

Antimicrobial class	Antibiotics	*E. faecalis* (*n* = 17)			*E. faecium* (*n* = 29)			*E. gallinarum* (*n* = 6)			*E. casseliflavus* (*n* = 2)			*E. hirae* (*n* = 3)			*E. durans* (*n* = 2)			Total (*n* = 59)		
		R	I	S	R	I	S	R	I	S	R	I	S	R	I	S	R	I	S	R	I	S
Penicillins	PEN	100	0	0	82.8	0	17.2	66.7	0	33.3	100	0	0	100	0	0	100	0	0	88.1	0	11.9
	PTZ	41.2	29.4	29.4	37.9	34.5	27.6	33.3	50	16.7	0	50	50	66.7	0	33.3	50	0	50	38.9	32.2	28.8
Lincosamides	CLI	70.6	29.4	0	72.4	10.4	17.2	33.3	16.7	50	50	0	50	66.7	33.3	0	100	0	0	67.8	16.9	15.3
Macrolides	ERY	52.9	47	0	62.1	27.6	10.4	33.3	66.7	0	0	100	0	0	100	0	0	100	0	49.2	45.8	5.1
Glycopeptides	VAN	47.1	17.7	35.3	44.8	31.1	24.1	16.7	50	33.3	0	0	100	0	0	100	0	50	50	37.3	27.1	36.6
	TEC	58.8	29.4	11.8	62.1	10.4	27.6	33.3	0	66.7	50	50	0	33.3	0	66.7	0	0	100	54.2	12.3	30.5
Tetracyclines	TET	52.9	17.7	29.4	51.7	27.6	20.7	33.3	16.7	50	0	50	50	33.3	66.7	0	0	50	50	45.8	27.1	27.1
	TGC	64.7	11.8	23.5	62.1	13.8	24.1	33.3	16.7	50	0	0	100	33.3	0	66.7	0	0	100	54.2	11.9	33.9
Aminoglycosides	KAN	17.7	5.8	76.4	10.4	20.7	68.9	0	0	100	0	0	100	0	0	100	0	0	100	10.2	11.9	77.9
Carbapenems	IMI	17.7	0	82.3	10.4	31.1	58.6	0	0	100	0	0	100	0	33.3	66.7	0	0	100	10.2	18.9	72.9
Quinolones	NOR	41.1	17.6	41.1	10.4	6.9	82.8	0	0	100	0	0	100	0	0	100	0	0	100	16.9	8.5	74.6
	CIP	47.1	11.8	41.1	41.4	3.5	55.2	16.7	0	83.3	0	0	100	0	0	100	0	0	100	35.6	5.1	59.3
Phenicols	CHL	23.5	58.8	17.7	24.1	41.4	34.5	33.3	66.7	0	0	100	0	0	100	0	0	100	0	22.0	55.9	22.0
Ansamycins	RIF	29.4	0	70.5	17.2	3.5	79.3	16.7	16.7	66.7	0	0	100	0	0	100	0	0	100	18.6	3.4	77.9

*PEN, penicillin G; PTZ, piperacillin; CLI, clindamycin; ERY, erythromycin; VAN, vancomycin; TEC, teicoplanin; TET, tetracycline; TGC, tigecycline; KAN, kanamycin; IMI, imipenem; NOR, norfloxacin; CIP, ciprofloxacin; CHL, chloramphenicol; RIF, rifampin.*

### Multiple Antibiotic Resistance Profile and Antibiotic Resistance Determinants

The multiple drug resistance (MDR) profile of the *Enterococcus* species revealed resistance to a minimum of three antibiotics that belong to three antimicrobial classes with a MARI of 0.21 and a maximum of 14 antibiotics that belong to 10 antimicrobial classes with a MARI of 1. A total of 37 (62.7%) isolates were multidrug resistant (Table 5). The occurrence of antibiotic resistance elements detected in Table 5 from the phenotypically resistant enterococci isolates to the macrolide, glycopeptide, and tetracycline antibiotics is as follows: *erm*A, 13/29 (44.8%); *mph*C, 13/29 (44.8%); *van*A, 14/22 (63.6%); *tet*A, 14/27 (51.9%); *tet*M, 15/27 (55.6%); *erm*B, 4/29 (13.8%); and *van*B, 5/22 (22.7%). None of *van*C-1, *van*C-2/3, *tet*B, *tet*C, and *tet*D was detected.

**Table 5:** Multidrug resistance profile, resistance phenotype, and resistance determinants of *Enterococcus* species from shrimps.

Isolate code	*Enterococcus* sp.	Shrimp variety	Biofilm formation	No. of antimicrobial class	No. of antibiotics	Resistance phenotype	MAR index	Resistance gene
ESM-1	*E. faecium*	Sauced	Moderate	6	7	PEN^R^-CLI^R^-ERY^R^-VAN^R^-TGC^R^-TEC^R^-RIF^R^	0.50	*erm*A-*mph*C-*van*A-*tet*A-*tet*M
ESM-2	*E. faecalis*	Boiled	Strong	3	4	PEN^R^-CLI^R^-TGC^R^-TEC^R^	0.29	
ESM-3	*E. casseliflavus*	Smoked	Negative	1	1	PEN^R^	0.07	
ESM-4	*E. faecium*	Sauced	Moderate	7	8	PEN^R^-KAN^R^-TET^R^-CLI^R^-CIP^R^-VAN^R^-TGC^R^-TEC^R^	0.57	*tet*A
ESM-5	*E. faecalis*	Smoked	Moderate	1	1	PEN^R^	0.07	
ESM-6	*E. casseliflavus*	Smoked	Moderate	3	3	PEN^R^-CLI^R^-TEC^R^	0.21	
ESM-7	*E. faecalis*	Sauced	Strong	8	12	PEN^R^-PTZ^R^-IMI^R^-TET^R^-CLI^R^-ERY^R^-CIP^R^-VAN^R^-TGC^R^-TEC^R^-RIF^R^-NOR^R^	0.86	*erm*A-*erm*B-*mph*C-*tet*A
EUMM-1	*E. faecalis*	Smoked	Negative	1	1	PEN^R^	0.07	
EUMM-2	*E. faecium*	Sauced	Moderate	8	11	PEN^R^-KAN^R^-TET^R^-CLI^R^-ERY^R^-CIP^R^-VAN^R^-TGC^R^-TEC^R^-NOR^R^-CHL^R^	0.79	*erm*A-*erm*B-*mph*C-*van*A-*tet*A-*tet*M
EUMM-3	*E. faecium*	Smoked	Negative	6	9	PEN^R^-PTZ^R^-TET^R^-CLI^R^-ERY^R^-CIP^R^-VAN^R^-TGC^R^-TEC^R^	0.64	*mph*C-*van*A
EUMM-4	*E. hirae*	Sauced	Negative	1	1	PEN^R^	0.07	
EUMM-5	*E. faecalis*	Boiled	Weak	4	4	PEN^R^-CLI^R^-TGC^R^-TEC^R^	0.29	
EUMM-6	*E. faecium*	Smoked	Moderate	2	2	PEN^R^-CLI^R^	0.14	
EUMM-7	*E. faecium*	Sauced	Weak	10	14	PEN^R^-PTZ^R^-IMI^R^-KAN^R^-TET^R^-CLI^R^-ERY^R^-CIP^R^-VAN^R^-TGC^R^-TEC^R^-RIF^R^-NOR^R^-CHL^R^	1	*erm*A-*van*A-*van*B-*tet*A-*tet*M
EUMM-8	*E. faecium*	Smoked	Negative			–	–	
EUMM-9	*E. faecium*	Smoked	Moderate	2	2	PEN^R^-CLI^R^	0.14	
EOMA-1	*E. faecalis*	Sauced	Moderate	7	11	PEN^R^-PTZ^R^-KAN^R^-TET^R^-CLI^R^-ERY^R^-CIP^R^-VAN^R^-TGC^R^-TEC^R^-NOR^R^	0.79	*erm*A-*mph*C-*van*A-*van*B-*tet*A
EOMA-2	*E. faecalis*	Boiled	Strong	8	12	PEN^R^-PTZ^R^-IMI^R^-TET^R^-CLI^R^-ERY^R^-CIP^R^-VAN^R^-TGC^R^-TEC^R^-NOR^R^-CHL^R^	0.86	*erm*A-*mph*C-*tet*M
EOMA-3	*E. faecium*	Smoked	Weak	1	1	PEN^R^	0.07	
EOMA-4	*E. faecium*	Sauced	Moderate			–	–	
EOMA-5	*E. faecium*	Smoked	Moderate	6	7	PEN^R^-CLI^R^-ERY^R^-VAN^R^-TGC^R^-TEC^R^-RIF^R^	0.50	*erm*A
EOMA-6	*E. durans*	Smoked	Negative	2	2	PEN^R^-CLI^R^	0.14	
EOMA-7	*E. faecium*	Boiled	Negative	7	10	PEN^R^-PTZ^R^-TET^R^-CLI^R^-ERY^R^-CIP^R^-VAN^R^-TGC^R^-TEC^R^-RIF^R^	0.71	*erm*A-*tet*A
EOMA-8	*E. gallinarum*	Smoked	Moderate	6	8	PEN^R^-PTZ^R^-TET^R^-CLI^R^-ERY^R^-TGC^R^-TEC^R^-CHL^R^	0.57	*tet*M
EAMA-1	*E. gallinarum*	Smoked	Negative	1	1	PEN^R^	0.07	
EAMA-2	*E. faecium*	Smoked	Negative	5	5	PEN^R^-CLI^R^-ERY^R^-TGC^R^-TEC^R^	0.36	
EAMA-3	*E. faecalis*	Sauced	Weak	7	10	PEN^R^-PTZ^R^-IMI^R^-KAN^R^-TET^R^-CIP^R^-VAN^R^-TGC^R^-NOR^R^-CHL^R^	0.71	*van*A-*tet*A
EAMA-4	*E. faecium*	Smoked	Moderate	5	6	PEN^R^-TET^R^-CLI^R^-ERY^R^-TGC^R^-TEC^R^	0.43	*mph*C-*tet*M
EAMA-5	*E. faecium*	Smoked	Strong	1	1	PEN^R^	0.07	
EAMA-6	*E. faecalis*	Sauced	Weak	6	6	PEN^R^-CLI^R^-ERY^R^-TGC^R^-TEC^R^-RIF^R^	0.43	*erm*A-*erm*B
EAMA-7	*E. faecium*	Smoked	Negative	1	1	PEN^R^	0.07	
EAMA-8	*E. faecium*	Sauced	Moderate	5	7	PEN^R^-PTZ^R^-TET^R^-CLI^R^-ERY^R^-TGC^R^-TEC^R^	0.50	*mph*C-*tet*A
EAMA-9	*E. gallinarum*	Smoked	Moderate			–	–	
EAMA-10	*E. faecium*	Sauced	Moderate	8	11	PEN^R^-PTZ^R^-IMI^R^-TET^R^-CLI^R^-ERY^R^-CIP^R^-VAN^R^-TGC^R^-TEC^R^-CHL^R^	0.79	*mph*C-*van*A
EIMW-1	*E. faecium*	Sauced	Strong	7	9	PEN^R^-PTZ^R^-IMI^R^-TET^R^-CLI^R^-ERY^R^-CIP^R^-TGC^R^-TEC^R^	0.64	*tet*M
EIMW-2	*E. faecium*	Sauced	Negative	3	3	TET^R^-CLI^R^-ERY^R^	0.21	
EIMW-3	*E. faecalis*	Sauced	Weak	1	1	PEN^R^	0.07	
EIMW-4	*E. faecalis*	Smoked	Weak	9	12	PEN^R^-KAN^R^-TET^R^-CLI^R^-ERY^R^-CIP^R^-VAN^R^-TGC^R^-TEC^R^-RIF^R^-NOR^R^-CHL^R^	0.86	*erm*A-*van*A-*van*B-*tet*A-*tet*M
EIMW-5	*E. faecium*	Smoked	Moderate	1	1	PEN^R^	0.07	
EIMW-6	*E. faecium*	Smoked	Moderate	4	5	PEN^R^-TET^R^-CLI^R^-TGC^R^-TEC^R^	0.36	*tet*M
EIMW-7	*E. faecium*	Sauced	Weak	2	2	PEN^R^-ERY^R^	0.14	
EIMW-8	*E. faecalis*	Boiled	Weak	3	3	PEN^E^-CLI^R^-ERY^R^	0.21	
EIMW-9	*E. faecium*	Smoked	Moderate	7	11	PEN^R^-PTZ^R^-TET^R^-CLI^R^-ERY^R^-CIP^R^-VAN^R^-TGC^R^-TEC^R^-NOR^R^-CHL^R^	0.79	*van*A-*tet*M
EIMW-10	*E. faecium*	Sauced	Negative	6	9	PEN^R^-PTZ^R^-TET^R^-CLI^R^-ERY^R^-CIP^R^-VAN^R^-TGC^R^-TEC^R^	0.64	*erm*A-*tet*M
EIMW-11	*E. faecalis*	Smoked	Negative	8	11	PEN^R^-PTZ^R^-TET^R^-CLI^R^-ERY^R^-CIP^R^-VAN^R^-TGC^R^-TEC^R^-RIF^R^-CHL^R^	0.79	*van*A-*van*B-*tet*A
EIMW-12	*E. gallinarum*	Sauced	Negative	1	1	PEN^R^	0.07	
EIMW-13	*E. gallinarum*	Sauced	Moderate			–	–	
EIMW-14	*E. faecium*	Sauced	Strong			–	–	
MOI-1	*E. faecalis*	Smoked	Negative	1	1	PEN^R^	0.07	
MOI-2	*E. faecalis*	Smoked	Weak	6	10	PEN^R^-PTZ^R^-TET^R^-CLI^R^-ERY^R^-CIP^R^-VAN^R^-TGC^R^-TEC^R^-NOR^R^	0.71	*erm*A-*erm*B-*mph*C-*van*A-*tet*M
MOI-3	*E. faecium*	Sauced	Negative	8	11	PEN^R^-PTZ^R^-TET^R^-CLI^R^-ERY^R^-CIP^R^-VAN^R^-TGC^R^-TEC^R^-RIF^R^-CHL^R^	0.79	*van*A-*van*B-*tet*A-*tet*M
MOI-4	*E. durans*	Smoked	Moderate	3	3	PEN^R^-PTZ^R^-CLI^R^	0.21	
MOI-5	*E. faecium*	Sauced	Negative	7	9	PEN^R^-PTZ^R^-CLI^R^-ERY^R^-CIP^R^-VAN^R^-TGC^R^-TEC^R^-CHL^R^	0.64	*mph*C
MOI-6	*E. faecium*	Sauced	Moderate	7	10	PEN^R^-PTZ^R^-TET^R^-CLI^R^-ERY^R^-CIP^R^-VAN^R^-TGC^R^-TEC^R^-CHL^R^	0.71	*erm*A-*van*A-*tet*A-*tet*M
MOI-7	*E. faecalis*	Boiled	Moderate	3	3	PEN^R^-TET^R^-CLI^R^	0.21	
MOI-8	*E. faecalis*	Sauced	Strong	8	11	PEN^R^-PTZ^R^-TET^R^-CLI^R^-ERY^R^-CIP^R^-VAN^R^-TGC^R^-TEC^R^-RIF^R^-NOR^R^	0.79	*mph*C
MOI-9	*E. hirae*	Sauced	Negative	4	6	PEN^R^-PTZ^R^-TET^R^-CLI^R^-TGC^R^-TEC^R^	0.43	
MOI-10	*E. hirae*	Smoked	Negative	2	3	PEN^R^-PTZ^R^-CLI^R^	0.21	
MOI-11	*E. gallinarum*	Sauced	Weak	8	11	PEN^R^-PTZ^R^-TET^R^-CLI^R^-ERY^R^-CIP^R^-VAN^R^-TGC^R^-TEC^R^-RIF^R^-CHL^R^	0.79	*mph*C-*van*A-*tet*A-*tet*M

*ESM, *Enterococcus* from Sapele market; EUMM, *Enterococcus* from Ughelli main market; EOMA, *Enterococcus* from Ogbegonogo market, Asaba; EAMA, *Enterococcus* from Ashafor market, Aniocha; EIMW, *Enterococcus* from Igbudu market, Warri; MOI, *Enterococcus* from main market, Oleh, Isoko; PEN, penicillin G; PTZ, piperacillin; CLI, clindamycin; ERY, erythromycin; VAN, vancomycin; TEC, teicoplanin; TET, tetracycline; TGC, tigecycline; KAN, kanamycin; IMI, imipenem; NOR, norfloxacin; CIP, ciprofloxacin; CHL, chloramphenicol; RIF, rifampin.*

A total of 19/26 (73.1%) isolates from sauced shrimps were MDR; all 6/6 (100%) isolates from boiled shrimp were MDR while 12/27 (44.4%) isolates from smoked shrimps were MDR. The relationship between antibiotic resistance and biofilm formation in this study is as follows: moderate biofilm formation (resistant between 0 and 11 antibiotics), weak biofilm formation (resistant between 1 and 14 antibiotics), strong biofilm formation (resistant between 1 and 12 antibiotics), and negative biofilm formation (resistant between 0 and 11 antibiotics; Table 5).

## Discussion

Enterococci make up the most predominant bacteria group that occur in foods, particularly as a consequence of their resistance to severe conditions in the environment during production technology, coupled with their high adaptability and conditions of food storage. Studies have reported enterococci incidence of seafood origin. Several studies have reported seafood contamination to naturally occur from an environment where fish are usually collected. Cross-contamination can occur as a result of food preparation or processing where bacteria are conveyed from utensils or from contaminated surfaces to seafood that are hygienically safe (Shikongo-Nambabi, 2011). When shrimps are harvested, they are usually processed by washing and exported frozen. During washing and freezing, decrease in the levels of bacteria can occur. However, resistant survivors can adulterate the final product traded at an open market, which can be distributed to consumers (Yano et al., 2011). An overall prevalence of 59 (8.19%) was recorded from the shrimps in this study. The prevalence of *E. faecalis* from cultured Indian prawn at Damietta governorate, Egypt, was 7% (El-Far et al., 2015), which was similar to the findings of this study. However, higher prevalence within the range of 16.7–74.1% has been documented previously (Koluman et al., 2009; Jamet et al., 2012; Jahan et al., 2013; Pesavento et al., 2014; Boss et al., 2016; Chajecka-Wierzchowska et al., 2016; Mus et al., 2017; Naas et al., 2017).

Higher incidence of *E. faecium* was recovered from the *Enterococcus* species in this study. This contradicts the findings from previous studies that reported *E. faecalis* as the most predominant enterococci from food sampled (Hammad et al., 2014; Pesavento et al., 2014; Chajecka-Wierzchowska et al., 2016). Similarly, investigations carried out in Tunisia (Belgacem et al., 2010) and Portugal (Barbosa et al., 2014) also revealed contamination of fermented meat products principally with *E. faecium* and *E. faecalis* isolates. Mus et al. (2017) did not isolate *E. gallinarum* from their study; however, Jahan et al. (2013) isolated *E. gallinarum* from meat and fermented meat products, which coincide with the findings from this study. PCR detection of five species isolated from RTE food samples tested by Chajecka-Wierzchowska et al. (2016) includes *E. faecium, E. hirae, E. faecalis, E. casseliflavus* and *E. durans*, with *E. faecium* (39.7%) and *E. faecalis* (48.7%) accounting for majority of the strains. This was in line with the findings of this study.

Literature has connected virulence in enterococci to diverse factors, such as *esp, gel*E, *ace, ccf, cyl*L, *cpd, agg, cob*, and *efa*A and formation of biofilm (Chuang et al., 2009). PCR detection of these genes coding virulence showed distinctiveness in virulence between enterococci. *E. faecalis* and *E. faecium* strains harbored extra virulence elements compared to other species, which was in line with findings from previous studies (Martin et al., 2005; Han et al., 2011). Barbosa et al. (2014) explained that there were no virulence genes detected in *E. faecium* isolates with the detection of the *esp, efa, gel*E, and *agg* genes in *E. faecalis*, which contradicts the findings of this study. Jahan and Holley (2014) reported the detection of numerous virulence elements in *E. faecium* (*esp, efa, agg*, and *gel*E) and *E. faecalis* (*esp, efa, gel*E, *ace*, and *agg*) strains recovered from meat and meat products. In addition, Jahan and Holley (2014) observed the *ace* gene in one *E. gallinarum* isolate. Belgacem et al. (2010) revealed that *E. faecium* strains were positive for *efa* and *gel*E genes. These were in line with the findings of this study.

Tsikrikonis et al. (2012) reported that the *gel*E gene is responsible for gelatinase production. Gelatinase is a metalloproteinase that can cleave hemoglobulin, insulin, casein, collagen, gelatin, and fibrinogen, in addition to numerous peptides/proteins (Giridhara-Upadhyaya et al., 2010). The findings from this study revealed that all enterococci positive for the *gel*E gene produced the enzyme phenotypically. Marra et al. (2007) reported that occurrence of the *gel*E gene is not particularly linked to gelatinase production, since Lindenstrau et al. (2011) suggest that other elements can be ascribed to *gel*E expression phenotypically. All *cyl*L gene-carrying isolates in this study expressed β-hemolytic activity. However, not all the isolates that phenotypically expressed the β-hemolytic activity harbored the *cyl*L gene. A contrasting situation was reported previously where some cytolysin gene-carrying isolates did not express β-hemolytic activity phenotypically (Eaton and Gasson, 2001; Togay et al., 2010).

Most enterococci strains from this study harbored the *esp* and *efa*A genes. Both genes appear to add to the persistence and colonization of enterococci in urinary tract infections (UTIs). The genes *ccf, cob*, and *cpd* (sex pheromone elements) were likewise detected in significant amounts of enterococci strains. Sex pheromone elements were identified solely in strains of *E. faecalis*, with all strains of *E. faecium* clear of the sex pheromone genes (Eaton and Gasson, 2001). This was not in agreement with our findings as a minimum of two out of the three sex pheromone elements were detected in all species tested and in line with the findings of Chajecka-Wierzchowska et al. (2016). Pheromone genes are occasionally followed with, and other times deprived of, the *agg* element (Eaton and Gasson, 2001). The S-layer and *ace* are surface proteins with adherence characteristics. The *ace* factor plays a significant role in enterococci establishment via adherence to the extracellular protein matrix. It also partakes in adhering type I and type IV collagen together (Nallapareddy et al., 2000). It has been reported in the literature via molecular methods that genes homologous to *efa*A occur in strains of *Enterococcus asini, Enterococcus avium, Enterococcus solitarius*, and *E. durans* (Semedo et al., 2003; Jimenez et al., 2013).

The *esp* gene is situated on the pathogenicity island and similarly comprises proteins that are liable for the dynamics of antibiotic release (Leavis et al., 2004). Literature reports on the *esp* protein established its involvement in biofilm and its role as a significant mechanism in exchanging genetic determinants inherently and elevating their antibiotic resistance characteristics (Foulquie-Moreno et al., 2006; Latasa et al., 2006). The occurrence of the *esp* gene in *E. faecium* has been reported to correlate imipenem, ampicillin, and ciprofloxacin resistance (Billstrom et al., 2008). Current reports propose a correlation of the occurrence of resistance to vancomycin and surface protein. Ochoa et al. (2013) reported that nosocomial strains have revealed that 83.3% of vancomycin-resistant *E. faecium* were positive for the *esp* gene, which was similar to the findings of this study where all the enterococci isolates that were vancomycin resistant harbored the *esp* gene. Most of the enterococci that simultaneously harbored the *esp* genes with vancomycin resistance by Billstrom et al. (2008) were multidrug resistant, which was also similar to the findings from our study. Oancea et al. (2004) reported that the *esp* element can be disseminated within strains of *E. faecalis* via the chromosome–chromosome transposition as well as within strains of *E. faecium* via conjugation of plasmid.

Hyaluronidase enzyme is crucial in breaking down mucopolysaccharides of the cartilage and connective tissue resulting in bacteria spread. The *hyl* element harboring isolates of nosocomial origin has been established to be frequent in *E. faecium* and rarely occurring in *E. faecalis* (Vankerckhoven et al., 2004). In addition, the *hyl* determinant has also been detected in other *Enterococcus* species such as *E. durans, E. mundtii*, and *E. casseliflavus* recovered from food (Trivedi et al., 2011). The aggregation substance (*agg* gene) being a virulence factor conveys antibiotic resistance determinants (Chajecka-Wierzchowska et al., 2017). The aggregation substance encompasses diverse adhesins, coded on conjugative plasmids conveyed in an expedited conjugation pathway, intermediated by sex pheromones determinants (Strzelecki et al., 2011). Clewell et al. (2000) described sex pheromones as small, non-water-loving peptides, which goes into aggregation substance and intermingle with a definite conjugative plasmid. The mechanism is essential in the dissemination of determinants among cells. Aggregation substance functions in the propagation within a particular plasmid, where factors encode virulence such as antibiotic resistance elements and cytolysin determinants. Cytolysin and aggregation elements can function together at the same time, elevating virulence by activating cytolysin regulation within the quorum-sensing pathway, thus conceivable to harm innermost tissues (Gilmore et al., 2002; Foulquie-Moreno et al., 2006).

A significant factor in enterococci pathogenesis is the formation of biofilm. Creti et al. (2004) reported that the higher amount of biofilm formed among *E. faecalis* compared to other *Enterococcus* species is not dependent on their source. Previous studies have reported that biofilm formation is not dependent on the presence or absence of the *esp* gene (Hufnagel et al., 2004; Kristich et al., 2004; Mohamed et al., 2004) while other researchers have stated clearly that there exists a positive correlation between biofilm formation and the occurrence of *esp* (Tendolkar et al., 2004; Chuang-Smith et al., 2010). Hancock and Perego (2004) reported that gelatinase production/determinant has also been reported to mediate signals that arrive through the quorum-sensing *fsr* system leading to biofilm formation. Mohamed and Murray (2005) documented that no relationship exists between biofilm and gelatinase activity in a significant amount of *E. faecalis* isolates. Serine protease (*spr*E) encodes enzymes that hydrolyze peptide bonds in proteins, in which case serine serves as the nucleophilic amino acid at the active site. Mohamed et al. (2004) stated that serine protease was more vital than gelatinase in biofilm production. Findings from our study revealed that the occurrence of *gel*E and *esp* elements was independent of biofilm formation, which was also in line with those of Chajecka-Wierzchowska et al. (2016).

The most prevailing issue of enterococci includes resistance to antibiotics used as therapeutics in treating human patients. This is of particular interest since 54 (91.5%) enterococci from this study were resistant to ≥1 antibiotics, with a total of 37 (62.7%) enterococci isolates being multidrug resistant. Multiple antibiotic resistances to ≥4 antibiotics were reported in enterococci from abattoir and aquaculture environs (Akinbowale et al., 2006; Igbinosa et al., 2017). The relatively high percentage of MDR enterococci recovery from RTE shrimps portrays them as an important reservoir of antibiotic resistance. Lack of antibiotic sensitivity to subclasses and classes of antibiotics such as quinolones and fluoroquinolones could result in difficulty in handling enterococci infections. Isolates recovered from RTE shrimp samples in this study resisted ciprofloxacin (used in the treatment regimen of meningitis and pneumonia) and norfloxacin (used in the treatment regimen of UTI as well as in infections as a consequence of enterococci that resisted vancomycin with bacteremia inclusive). Resistance to quinolone antibiotics was observed solely in *E. faecalis, E. gallinarum*, and *E. faecium*. The proportion of quinolone-resistant enterococci recovered from RTE shrimp calls for concern, in view of the astonishing capacity of enterococci to disseminate resistance.

The highest frequency of resistance by Mus et al. (2017) was observed for tetracycline (21.7%), followed by ciprofloxacin (2.0%), penicillin (2.0%), and ampicillin (1.0%), which were lower compared to our finding. *E. faecalis* revealed a higher prevalence of antibiotic resistance than other enterococci as described by Mus et al. (2017), which was in line with the findings of this study. The percentage of MDR enterococci by Mus et al. (2017) was 3.4%, which was lower than the findings of this study. An overall total of 54 (91.5%) isolates in our study that harbored a virulence gene were simultaneously resistant to a minimum of one antibiotic. All *E. durans, E. casseliflavus, E. hirae*, and *E. faecalis* in our study simultaneously harbor a virulent trait and were resistant to at least one antibiotic while 3 (10.4%) *E. faecium* and 2 (33.3%) *E. gallinarum* only harbor a virulent trait and completely sensitive to all antibiotics tried. This contradicts the study by Mus et al. (2017) where *E. faecalis* 29 (26.6%), which carries one of the virulence-associated determinants, were simultaneously resistant to a minimum of one antibiotic. All enterococci strains screened for their antibiotic-resistant profiles revealed high multiresistance phenotype (Naas et al., 2017), which was contradicted by the findings from this study. Species-specific primers by Mus et al. (2017) revealed *E. faecalis* as the predominant *Enterococcus* species carrying one or more virulence-associated determinants. Although all *E. faecalis* from this study harbored ≥3 virulence elements in this study, *E. faecium* was the most predominant of the *Enterococcus* species.

Boss et al. (2016) reported that 16% of *E. faecalis* of sampled shrimp imported into Switzerland were resistant to tetracycline. A significant proportion of enterococci by Chajecka-Wierzchowska et al. (2016) revealed that a significant proportion of the isolates were resistant to erythromycin (42.7%). Significant level of erythromycin resistance was also witnessed in this study. Enterococci are particularly resistant intrinsically (Chow, 2000). Intrinsic mechanisms usually result in decreased level of resistance while acquirement of mobile determinants particularly underscores a high level of resistance. The findings by Arumugam et al. (2017) showed that *E. faecalis* from seafood possess multiple antibiotic resistances. These reports further consolidate the findings from this study. The dynamics and emergence of antimicrobial resistance determinants in bacteria that circulate between the environment, humans, and animals are not completely known. Selective pressure resulting from antimicrobials on the microbiomes of human and animal and their environments (particularly healthcare institutions and farms) as well as soil and sewage systems can likely confer persistence benefits on bacteria with antimicrobial resistance elements, which may further be spread via integrons, plasmids, or transposons (Cheng et al., 2015).

Application of antibiotics as growth promoters is prohibited in Europe as far back as 2006. They are frequently applied in veterinary medicine for prophylactic purposes and as a treatment regimen. Antibiotics that are used as therapeutics for infections in humans might not be applied but can be important in the selection of resistant enterococci. Tetracyclines are usually applied as they are permitted by the Food and Drug Administration and the European Union for use in treating gastritis and hepatitis; diseases of the urinary, reproductive, and respiratory system; and bacterial infections that emanate from the skin (Martinez, 2009). Tetracycline antibiotics are used in swine, cattle, poultry, sheep, fish, and goats (Bea-Ven et al., 2014). Its application in veterinary medicine has resulted in an upsurge in the number of antibiotic-resistant strains and selective pressure. It has also been documented that tetracycline-resistant enterococci usually portray combined resistance to aminoglycoside (gentamicin) and, to some extent, glycopeptide (vancomycin) (Choi and Woo, 2014). High occurrence of tetracycline resistance has previously been documented among enterococci strains from divergent sources (Templer and Baumgartner, 2007; Chajecka-Wierzchowska et al., 2012). The occurrence of MDR enterococci isolates in the food chain is of a significant concern due to the ease of resistance gene dissemination to other bacteria. Enterococci have been described as a major concern in the last decades, as they have become one of the most vital nosocomial infections that cause serious illnesses in humans. The occurrence of *Enterococcus* spp. in seafood may act as gene pools of antibiotic resistance determinants (Valenzuela et al., 2009). Chajecka-Wierzchowska et al. (2016) reported that enterococci extensively occur in retail RTE meat or meat products with lots of enterococci strains such as *E. durans, E. casseliflavus, E. gallinarum*, and *E. hirae* harboring antibiotic resistance and conveying exchangeable resistance determinants.

Vancomycin-resistant enterococci (VRE) have been described as clinical pathogens that have been recovered from environmental habitats. The dissemination of opportunistic pathogens that harbor vancomycin-resistant genes further than hospital environments into the community is an impending public health threat as vancomycin is referred to as the last line of defense against enterococci infections. A total of 35 VRE outbreaks have been systematically reviewed and documented by Ulrich et al. (2017), from which 757 patients were affected and 77 died; the prevalent site of pathogen recovery was rectal swabs or stool samples. The principal modes of documented spread were contact-to-contaminated environment, patient-to-patient, and hands of healthcare workers, while the predominant risk factor was previous antibiotic treatment regimen (Ulrich et al., 2017). The prevalent infection control procedures carried out by Ulrich et al. (2017) were screening and isolation patient cohort. Sivertsen et al. (2016) reported a vancomycin variable *van*A+ enterococci outbreak that deviated from phenotypic expression via Clinical Laboratory Standards Institute guidelines and demonstrated the molecular machineries for switching *in vivo* into vancomycin resistance with lateral dissemination of the *van*A cluster. Schwaiger et al. (2009) revealed that genetic elements have an impact on resistance levels. A cluster of the silenced *van*A gene from patients on an exchangeable plasmid resulted in an outbreak of variable vancomycin enterococci (Sivertsen et al., 2016).

Kümmerer (2009) reported that genetic elements are frequently recovered from environmental isolates compared to food isolates. Genes that harbor resistance to tetracyclines in significant numbers of enterococci are confined to transposons frequently compared to plasmids (Chajecka-Wierzchowska et al., 2016). Such genes are sited on a transposon close to the *erm*B element, often considered as the prevalent determinant coding macrolide resistance. Erythromycin is an antibiotic that belongs to the macrolide class and used in the therapeutic regimen of infection in humans, particularly those with a documented allergic history to penicillin. The pattern of antibiotic resistance comprises disruption of the antibiotic passage into cells, which alters the target site of the antibiotic. The *erm*B gene is usually detected in enterococci strains recovered from clinical, environmental, and food sources and other Gram-positive cocci such as *Staphylococcus aureus* (Ding et al., 2012), *Streptococcus pyogenes* (Palmieri et al., 2012), or *Streptococcus pneumoniae* (Reijtman et al., 2013). Aside from the *erm*B gene, *E. faecalis* and *E. faecium* from food samples had a significant range of elements, which code for *N*-6 methyltransferase (*erm*C; *erm*A) or those accountable for the enterococci efflux characteristics (*mef*A/E; *msr*C). It has been described that *erm*A is frequently recovered in phenotypically macrolide-resistant *S. aureus* and other staphylococci (Zmantar et al., 2011).

Antibiotic usage must be firmly controlled to eradicate selective pressure, including regulating the application of antibiotic in veterinary practice and human medicine and incorporation of antibiotics as growth enhancers in animal feed. Increased attentiveness to infection control procedures to decrease the risk of obtaining resistant enterococci is crucial, particularly during institutionalization in healthcare facilities or antimicrobial use. The cycle of dissemination must be interjected through environmental cleaning, proper hand hygiene, avoidance of raw or undercooked food, and compliance with infection control procedures by healthcare personnel, patients, and visitors, specifically in the course of treatment with antibiotics. Furthermore, practical microbiological screening of patients hospitalized with associated risk factors for conveying resistant bacteria, as well as history of transfer from other hospitals, prolonged hospitalization, and transfer to endemic countries; directly pragmatic hand hygiene prior to intake of oral drugs, drinks, and food; and specific disinfection of mutual-touch or high-touch items, such as bed curtains and bed rails, are imperative. Final eradication and containment of the epidemic clones were attained by environmental decontamination using hydrogen peroxide vapor, absolute isolation deterrents, a vancomycin-resistant confinement ward, and antimicrobial stewardship (Frakking et al., 2018).

Auto-inducing peptides have been reported to be part of the intercellular network in Gram-positive bacteria (Sturme et al., 2002). Majority of these peptides are released via posttranslational modification in different ways and dedicated systems and are lastly sensed by other cells through receptors located in the membrane that are part of two-component regulatory systems. In this mechanism, the expression of different functions including genetic competence, virulence, and production of antimicrobials can be moderated in a coordinated cell-density- and growth-phase-dependent manner. Wide and indiscriminate application of antibiotics has led to serious ecological and biological concerns, particularly the development of antibiotic resistance. Probiotics are being suggested as an eco-friendly and effective substitute to antibiotics (Zorriehzahra et al., 2016). Ethyl acetate and methanol root extract of *Anethum sowa* L. have been reported to show good antibacterial activity against *Enterococcus* species (Saleh-e-In et al., 2016). *Enterococcus lactis* strain has been demonstrated to display bacteriocin-like activities against Gram-positive bacteria (Braïek et al., 2017).

## Conclusion

This study revealed that *Enterococcus* species with biofilm potentials and extracellular virulence properties extensively occur in retail RTE shrimps. A significant number of isolated strains are resistant to antibiotics and harbor resistant and virulent genes, denoting a significant route of resistance and virulence dissemination to bacteria in humans. There is an inadequate understanding of the intricacies of antibiotic-resistant enterococci of food origin that belong to enterococci aside from *E. faecium* and *E. faecalis*. Findings from this study reveal detailed antibiotic resistance of *E. durans, E. casseliflavus, E. gallinarum*, and *E. hirae*. Finally, this study reveals that RTE seafood products are reservoirs of potential virulent enterococci with antibiotic-resistant capabilities that provide useful data for risk assessment and indicates that these foods may present a public health risk to consumers.

## Author Contributions

AB carried out the sampling, laboratory procedures, data interpretation, and writing of the manuscript. EI conceptualized, designed, and supervised the research, and contributed in the laboratory methodologies and data interpretation, as well as in the writing of the manuscript. Both authors have read and approved the manuscript.

## Conflict of Interest Statement

The authors declare that the research was conducted in the absence of any commercial or financial relationships that could be construed as a potential conflict of interest.
